# Disparities by Race and Ethnicity in Percutaneous Coronary Intervention

**DOI:** 10.1001/jamanetworkopen.2025.32660

**Published:** 2025-09-18

**Authors:** Charleen Hsuan, Michelle P. Lin, Alexis Zebrowski, Renee Y. Hsia, Brendan G. Carr, David J. Vanness, Eric K. Wei, David G. Buckler, Haoyu Bi, Jeannette A. Rogowski

**Affiliations:** 1 Department of Health Policy and Administration, Pennsylvania State University, University Park; 2 Department of Emergency Medicine, Stanford University, Palo Alto, California; 3 Department of Emergency Medicine, Icahn School of Medicine at Mount Sinai, New York, New York; 4 Mount Sinai Health System, New York, New York; 5 Department of Population Health Science and Policy, Icahn School of Medicine at Mount Sinai, New York, New York; 6 Department of Emergency Medicine, University of California, San Francisco, San Francisco; 7 Philip R. Lee Institute for Health Policy Studies, University of California, San Francisco, San Francisco; 8 NYC Health + Hospitals, New York, New York

## Abstract

**Importance:**

Hispanic and non-Hispanic Black patients with ST-segment elevation myocardial infarction (STEMI) are less likely than White non-Hispanic patients to receive guideline-recommended percutaneous coronary intervention (PCI). Research suggests disparities arise before and during STEMI treatment, but it is unclear when the largest disparities in PCI emerge.

**Objective:**

To assess when in the care process the largest disparities in PCI receipt occur in patients with STEMI presenting to an emergency department.

**Design, Setting, and Participants:**

This cross-sectional study evaluated adult patients with STEMI presenting to Florida hospitals from January 1, 2011, to December 31, 2021. Data were analyzed from June 29, 2023, to May 29, 2025.

**Exposure:**

Patient race and ethnicity.

**Main Outcomes and Measures:**

The main outcomes were presentation to PCI-capable hospitals, receipt of PCI if initially presenting to PCI-capable hospitals, transfer if initially presenting to non-PCI capable hospitals, and receipt of PCI at receiving hospital if transferred. Logistic regression was used to compare outcomes for patients with STEMI by race and ethnicity, controlling for payer, age, sex, weekend presentation, time of presentation, comorbidities, and hospital characteristics.

**Results:**

Among 139 629 patients with STEMI included in the analysis, 68.81% were male. Mean (SD) age was 64.4 (13.0) years. A total of 9.09% identified as Black, 15.17% as Hispanic, 70.56% as White, and 5.17% as other or missing race. In adjusted analyses, Black (−1.8 [95% CI, −2.6 to 1.1] percentage points [pp]) and Hispanic (−3.1 [95% CI, −3.7 to −2.4] pp) patients were less likely than White patients to present to PCI-capable hospitals (*P* < .001 for both). Among patients initially presenting to PCI-capable hospitals, Black patients were less likely to receive PCI than White patients (−8.6 [95% CI, −9.5 to −7.7] pp; *P* < .001). Among patients initially presenting to non–PCI-capable hospitals, Black (−4.0 [95% CI, −6.4 to −1.5] pp; *P* = .001) and Hispanic (−4.2 [95% CI, −6.3 to −2.0] pp; *P* < .001) patients were less likely to be transferred than White patients. Among transferred patients, Black patients were less likely to undergo PCI at the receiving hospital than White patients (−13.3 [95% CI, −16.6 to −9.9] pp; *P* < .001).

**Conclusions and Relevance:**

In this cross-sectional study examining racial and ethnic disparities in receipt of PCI for patients with STEMI, racial and ethnic disparities persisted throughout the care process. The largest magnitude of disparity was PCI receipt if transferred, but the disparity with the largest impact was PCI receipt when initially presenting to PCI-capable hospitals.

## Key Points

**Question:**

What hospital factors are associated with disparities by race and ethnicity in receipt of guideline-recommended percutaneous coronary intervention (PCI) among patients with ST-segment elevation myocardial infarction?

**Findings:**

In this cross-sectional study of 139 629 participants, Black and Hispanic patients were less likely than White patients to initially present to PCI-capable hospitals, receive PCIs if initially presenting to PCI-capable hospitals, be transferred if initially presenting to non–PCI-capable hospitals, and receive PCIs if transferred.

**Meaning:**

These findings suggest that 2 areas of focus could be standardizing hospital protocols at PCI-capable hospitals and improving hospital transfers.

## Teaser

This cross-sectional study evaluates hospital factors associated with racial and ethnic disparities in percutaneous coronary intervention (PCI) receipt among patients with ST-segment elevation myocardial infarction.

## Introduction

Although acute myocardial infarction (AMI) remains a leading cause of death in the US, AMI-related deaths have decreased during the past 2 decades, partly due to interventions such as thrombolytic therapy and percutaneous coronary intervention (PCI).^1^ However, non-Hispanic Black patients consistently experience the highest age-adjusted mortality rates; in 2019, age-adjusted mortality rates were 16.8% higher for Black patients than for White patients.^1^

About one-quarter of AMIs are ST-segment elevation myocardial infarctions (STEMI).^2,3^ Because primary PCI reduces mortality in patients with STEMI by about 30%,^4^ clinical guidelines recommend PCI for STEMI, when available, within 90 minutes of hospital presentation if patients initially present to PCI-capable hospitals^5^ and within 120 minutes if patients need to be transferred.^6^ However, compared with White patients, Black and Hispanic patients with STEMI are less likely to receive PCI and more likely to die.^7,8^ These findings underscore the urgency of pinpointing where policy interventions should be targeted to reduce racial and ethnic disparities in PCI use.

Similarly to disparities in AMI,^9,10,11^ previous research suggests that disparities emerge both before and during treatment for STEMI. Disparities prior to the initiation of care include Black and Hispanic patients with STEMI being more likely than White patients to live in communities without PCI-capable hospitals^12,13^ and less likely to present to PCI-capable hospitals.^14^ Disparities also emerge after care is initiated. Black and Hispanic patients wait longer in the hospital before receiving PCI^15,16^ and are more likely to present to hospitals of lower quality, that are less resourced, or that perform fewer PCIs.^16,17,18,19^

These findings complicate attempts to target policy interventions that reduce racial and ethnic disparities, as it is unclear where interventions would be most effective. For instance, if disparities in PCI occur because Black patients with STEMI are more likely to present to non–PCI-capable hospitals than White patients,^12,20,21^ policy should improve geographic access to PCI. Alternatively, if disparities occur because of differences in transfer,^9^ policymakers should improve transfer protocols or Medicare and other payers should tie reimbursement policies to appropriate transfer.^22,23^ To better inform these efforts, we followed up a population of patients with STEMI from initial hospital presentation through necessary transfers to examine when disparities in PCI use emerged and their relative magnitude.

## Methods

### Study Overview

This cross-sectional study was deemed exempt, with a waiver of informed consent, by the institutional review board of Pennsylvania State University owing to use of deidentified data. We followed the Strengthening the Reporting of Observational Studies in Epidemiology (STROBE) guideline.

The eFigure in Supplement 1 describes 4 processes where racial and ethnic disparities can appear. First, disparities could come from racial and ethnic differences in whether patients present to PCI-capable hospitals (process A). Second, if patients present to PCI-capable hospitals, there could be disparities in whether initial hospitals perform PCI (process B). Third, if patients do not initially present to PCI-capable hospitals, there could be differences in whether they are transferred to another hospital (process C). Finally, for patients who did not initially present to PCI-capable hospitals but were transferred, there could be differences in receipt of PCI at the receiving hospital (process D).

### Data, Setting, and Participants

This study aimed to identify racial and ethnic disparities in the processes described in eFigure 1 in Supplement 1 using hospital discharge data from Florida. We focused on a single state to avoid introducing heterogeneous state policies and differences in hospital transfer patterns.^22^ We chose Florida since it is a large state (the third most populous US state) with clear policies, including emergency medical services protocols and licensing of cardiac hospitals.

We merged statewide all-payer hospital discharge data from the Healthcare Cost and Utilization Project (HCUP) state inpatient database and state emergency department databases for Florida^24,25^ from January 1, 2010, to December 31, 2021, with prior year data on hospital characteristics from the American Hospital Association Annual Hospital Data (2009-2020). The Florida HCUP data contain all-payer data on inpatient and emergency department (ED) visits to nonfederal hospitals.

The sample was a census of all ED visits by adults with a primary diagnosis of STEMI to nonfederal hospitals from January 1, 2011, to December 31, 2021 (codes in eTable 1 in Supplement 1). Because the study examined the index hospital to which a patient with STEMI presented, we excluded visits that were transfers from another hospital (eFigure 2 in Supplement 1); transfers to another hospital were included. Transfers were defined as having a visit on the same or the next day to another acute care hospital.^26^

### Variables

The independent variable used for estimation was the HCUP-classified combined race and ethnicity variable. Hospitals are required to report race and ethnicity data but may vary in how they collect this information, resulting in data quality problems.^27,28,29,30^ We categorized race and ethnicity as non-Hispanic Black (hereafter Black), Hispanic, non-Hispanic White (hereafter White), and other or missing, which included American Indian or Alaska Native, Asian, and Native Hawaiian or Other Pacific Islander because of sample size.

The dichotomous outcome dependent variables were whether patients with STEMI initially presented to PCI-capable hospitals (process A), receipt of PCI if patients initially presented to PCI-capable hospitals (process B), transfer if patients initially presented to non–PCI-capable hospitals (process C), and receipt of PCI at receiving hospitals if patients were transferred (process D). Following prior literature, a hospital was PCI-capable if it performed at least 50 PCIs the prior year^31^ (eTable 1 in Supplement 1). In sensitivity analyses, we used a state licensing definition and lower thresholds.^31,32^

Visit covariates were primary payer, patient age, patient age squared, sex, weekday or weekend visit, time (hour) of visit, Charlson Comorbidity Index,^33^ patient distance from hospital, and year. Payer included no insurance, Medicaid, Medicare, private, and other or missing. Patients dually eligible for Medicaid and Medicare were classified as having Medicare coverage due to data limitations. Time of visit was 12 to 7 am, 8 am to 3 pm, and 4 to 11 pm. Patient distance from the hospital used straight-line distance^34^ from the centroid of patient zip code tabulation area to hospital latitude and longitude. Hospital covariates were size (<200, 200-500, or >500 beds), academic status, ownership (public, nonprofit, or for profit), hospital referral region, and urbanicity.

### Statistical Analysis

Data were analyzed from June 29, 2023, to May 29, 2025. Our goal was to assess racial and ethnic disparities in PCI for the processes described in eFigure 1 in Supplement 1. Using logistic regression with robust SEs, we first estimated an overall population-level model to examine racial and ethnic disparities for all outcomes. We used robust SEs to account for clustering by hospital and controlled for the aforementioned visit and hospital characteristics (eTable 2 in Supplement 1). To examine whether disparities were driven by payer,^14,35^ in secondary analyses, we restricted the sample to Medicare patients. In this Medicare-only model, the coefficients for race and ethnicity represent the racial and ethnic disparity for Medicare patients. We then examined disparities within the same hospital using a hospital fixed-effects model for all outcomes except initial presentation. In this analysis, the coefficients for race and ethnicity represent the difference between a Black patient and a White patient within the same hospital. For PCI use (processes B and D), we also estimated the overall, Medicare-only, and hospital fixed-effects models stratified by hospital ownership.

As odds ratios are not comparable between models,^36,37,38^ we generated marginal effect estimates and predicted probabilities.^39,40^ We report *P* values for the marginal effect estimates, with 2-sided *P* < .05 indicating statistical significance. In further analyses, we tested robustness of our results to account for potential biases. First, to test whether Black and Hispanic patients were more likely than White patients to present at times when hospitals are less likely to perform PCI, perhaps because of patient delay or differences in staffing,^41^ we stratified by ED registration time and day of the week.

Second, we examined racial and ethnic differences in receipt of alternative therapies (fibrinolysis or coronary artery bypass graft [CABG] surgery) among patients with STEMI who did not receive PCI. While timely PCI is usually preferred, fibrinolysis and CABG are sometimes warranted.^6^

Third, we varied the definition of PCI capable. Fourth, we excluded 2020 and 2021 because the COVID-19 pandemic disrupted ED transfers and hospital protocols. Fifth, we separately examined *International Classification of Diseases, Ninth Revision* (*ICD-9*) and *International Statistical Classification of Diseases and Related Health Problems, Tenth Revision (ICD-10*) data. Sixth, we included patients who died or were transferred since they were excluded from the main analysis. Seventh, we excluded patients who were discharged against medical advice (they were included in the main analysis to avoid sample bias^42,43^). Eighth, we excluded patients with diabetes because of differences in clinical treatment.^6^ Ninth, we excluded women, as they may have delayed presentation.^44^ Tenth, we stratified by hospital characteristics that could explain results, including rurality, size, and percentage of patients with STEMI receiving PCI.^45^ Finally, to examine whether an unobserved confounder explains our findings, we calculated an E-value.^46^ An unobserved confounder would need to be uncorrelated with covariates and associated with both race and ethnicity and PCI utilization by a risk ratio of at least the E-value to explain results.

## Results

The sample consisted of 139 629 patients diagnosed with STEMI who presented to an ED from 2011 to 2021 (mean [SD] age, 64.4 [13.0] years). Of these, 9.09% were Black, 15.17% were Hispanic, 70.56% were White, and 5.17% were other or missing race (Table). A total of 85.71% were admitted to the initial hospital and received a discharge diagnosis. A total of 66.87% of Hispanic patients received PCI compared with 60.61% of Black patients and 69.00% of White patients. Because of sample size, most visit and hospital characteristics differed for patients of different races and ethnicities. For instance, Black patients compared with White patients were more likely to have more comorbidities (26.87% vs 22.49%), to initially present to academic hospitals (55.48% vs 46.13%), and to present to larger hospitals (>500 beds: 36.52% vs 26.38%).

**Table.** Descriptive Statistics for ED Patients With STEMI^a^

Characteristic	Patients, %				
	Hispanic (n = 21 182 [15.17])	Non-Hispanic Black (n = 12 692 [9.09])	Non-Hispanic White (n = 98 536 [70.60])	Other or missing (n = 7219 [5.17])^b^	All (N = 139 629)
ED disposition					
Lost to follow-up or treated and released	5.35	5.87	3.56	3.91	4.06
Admitted as inpatient	83.18	82.94	86.32	89.69	85.71
Transferred	10.34	9.25	8.63	4.90	8.75
Died	1.12	1.94	1.49	1.50	1.48
Received PCI at initial hospital	66.87	60.61	69.00	74.87	68.22
Visit characteristics					
Age, mean (SD), y	63.2 (13.3)	59.3 (13.7)	65.5 (12.7)	61.6 (12.8)	64.4 (13.0)
Sex					
Female	27.96	35.46	31.81	24.71	31.19
Male	72.04	64.54	68.19	75.29	68.81
Payer					
Uninsured	18.48	17.52	11.17	19.49	13.28
Medicaid	11.95	13.92	4.76	7.54	6.83
Medicare	42.15	40.45	53.39	35.91	49.61
Private	25.43	23.12	26.96	33.45	26.71
Other or missing	1.99	4.98	3.72	3.62	3.56
Charlson Comorbidity Index					
0	60.20	53.76	55.11	62.04	56.12
1	20.63	19.37	22.40	20.72	21.77
≥2	19.17	26.87	22.49	17.23	22.11
Weekend visit	28.76	29.45	28.68	29.01	28.78
Admission time					
12-7 am	21.61	24.16	21.30	19.71	21.52
8 am-3 pm	45.06	41.50	46.10	46.43	45.54
4-11 pm	33.33	34.34	32.60	33.86	32.94
Hospital characteristics					
Academic	57.40	55.48	46.13	60.13	45.18
Belongs to a system	85.36	83.44	86.13	90.11	86.97
Ownership					
Public	17.31	20.45	14.30	9.45	15.07
Nonprofit	39.27	44.42	47.46	51.53	46.15
For-profit	43.42	35.12	38.23	39.02	38.78
Bed size					
<200	18.03	18.69	25.26	14.05	22.99
200-500	47.86	44.79	48.35	50.37	48.06
>500	34.11	36.52	26.38	35.59	28.95
NCHS urbanicity					
Large central metropolitan	63.21	40.92	24.73	41.56	32.91
Large fringe metropolitan	22.51	26.16	25.23	27.83	25.03
Medium metropolitan	11.80	25.69	36.97	24.24	31.47
Small metropolitan, micropolitan, or noncore	2.48	7.23	13.07	6.37	10.59

Abbreviations: ED, emergency department; NCHS, National Center for Health Statistics; PCI, percutaneous coronary intervention; STEMI, ST-segment elevation myocardial infarction.

^a^ Differences across races for all characteristics were *P* < .001 except for admission on a weekend.

^b^ Includes patients identified as American Indian or Alaska Native, Asian, Native Hawaiian or Other Pacific Islander, those missing a race and ethnicity, or those identified as other by the original data source.

### Initial Presentation to PCI-Capable Hospital

**Figure 1.**
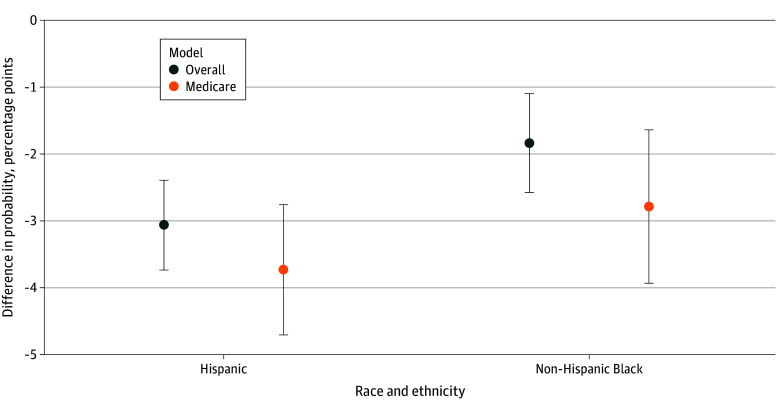
Difference in Probability of Presentation of Patients With ST-Segment Elevation Myocardial Infarction to a Percutaneous Coronary Intervention (PCI)–Capable Hospital Non-Hispanic White patients were the comparison group. *Overall* indicates the overall population-based model, which used robust SEs and controlled for visit and hospital characteristics. *Medicare* indicates the overall regression model in which the sample consisted only of Medicare patients. Other or missing race was excluded from the figure for clarity but included in the regression. Error bars indicate 95% CI.

In analyses adjusting for patient and hospital characteristics, 82.6% of White patients with STEMI presented to PCI-capable hospitals. Compared with White patients, Hispanic patients were 3.8% less likely (−3.1 [95% CI, −3.7 to −2.4] percentage points [pp]) and Black patients were 2.2% less likely (−1.8 [95% CI, −2.6 to 1.1] pp) to present to PCI-capable hospitals (*P* < .001 for both) Figure 1 and eTable 3 in Supplement 1) These differences were slightly larger among Medicare patients.

### Receipt of PCI Among Patients Initially Presenting to PCI-Capable Hospitals

**Figure 2.**
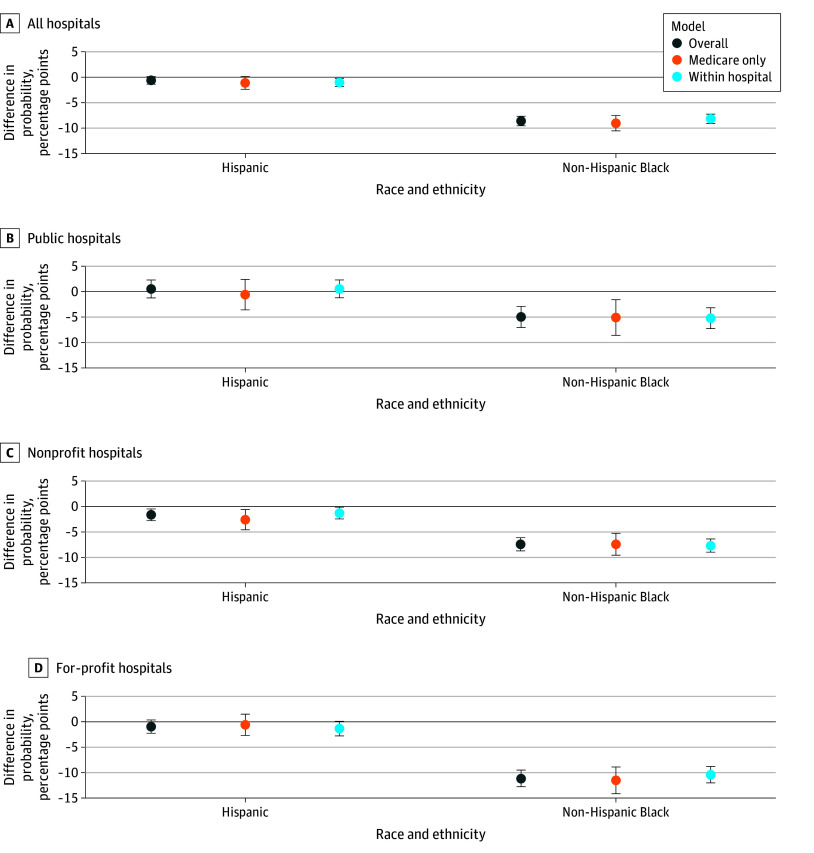
Difference in Probability of Receipt of Percutaneous Coronary Intervention (PCI) at the Initial Hospital When a Patient With ST-Segment Elevation Myocardial Infarction Initially Presented to a PCI-Capable Hospital Non-Hispanic White patients were the comparison group. *Overall* indicates the overall population-based model, which used robust SEs and controlled for visit and hospital characteristics. *Medicare only* indicates the overall regression model in which the sample consisted only of Medicare patients. *Within hospital* indicates a hospital fixed-effects model that controlled for visit characteristics and hospital fixed effects and indicates the difference compared with a non-Hispanic White patient within the same hospital. Other or missing race was excluded from the figure for clarity but included in the regression. Error bars indicate 95% CI.

Among patients initially presenting to PCI-capable hospitals who survived and were not transferred, 80.5% of White patients with STEMI received PCI after adjusting for patient and hospital characteristics. Compared with White patients, Black patients were 10.7% less likely (−8.6 [95% CI, −9.5 to −7.7] pp; *P* < .001) to receive PCI (Figure 2). These differences were similar in magnitude for patients within the same hospital and Medicare patients only. The difference between Black and White patients was present regardless of hospital ownership, with the smallest difference for public hospitals (−4.9 [95% CI, −7.1 to −2.9] pp) compared with nonprofit (−7.4 [95% CI, −8.7 to −6.1] pp) and for-profit (−11.2 [95% CI, −12.8 to −9.5] pp) hospitals (*P* < .001 for all). Most differences for Hispanic patients were not statistically significant.

In all sensitivity analyses, Black patients were less likely than White patients to receive PCI when they initially presented at PCI-capable hospitals. This difference appeared regardless of hour of presentation, weekend or weekday presentation, hospital bed size, PCI intensity, academic status, and urbanicity and when including men only, using *ICD-9* or *ICD-10* data only, excluding 2020 to 2021, excluding patients discharged against medical advice, excluding patients who had diabetes, and including patients who died or were transferred (eTable 4 in Supplement 1). Compared with White patients, Black patients who presented to PCI-capable hospitals were less likely to receive CABG and did not have differential receipt of fibrinolytics (eTable 5 in Supplement 1).

### Transfer if Presenting to a Non−PCI-Capable Hospital

**Figure 3.**
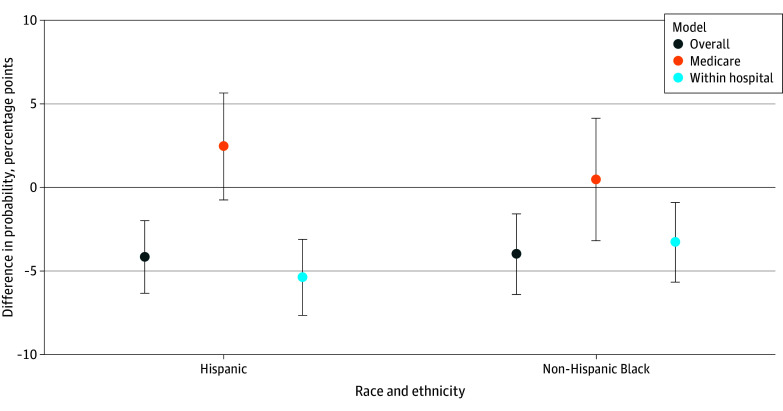
Probability of Transfer When a Patient With ST-Segment Elevation Myocardial Infarction Initially Presented to a Non–Percutaneous Coronary Intervention (PCI)–Capable Hospital Non-Hispanic White patients were the comparison group. *Overall* indicates the overall population-based model, which used robust SEs and controlled for visit and hospital characteristics. *Medicare only* indicates the overall regression model in which the sample consisted of Medicare patients only. *Within hospital* indicates a hospital fixed-effects model that controlled for visit characteristics and hospital fixed effects and indicates the difference compared with a White non-Hispanic patient within the same hospital. Other or missing race was excluded from the figure for clarity but included in the regression. Error bars indicate 95% CI.

In adjusted analyses, 74.8% of White patients who initially presented to a hospital without PCI capability were transferred to another acute care hospital (Figure 3). Compared with White patients, Hispanic patients were 5.6% less likely (−4.2 [95% CI, −6.3 to −2.0] pp; *P* < .001) and Black patients were 5.3% less likely (−4.0 [95% CI, −6.4 to −1.5] pp; *P* = .001) to be transferred. These differences were similar for Black patients (−3.3 [95% CI, −5.7 to −0.9] pp; *P* = .005) and Hispanic patients (−5.4 [95% CI, −7.7 to −3.1] pp; *P* < .001) within the same hospital. Differences for Medicare patients only were not statistically significant.

The racial and ethnic disparities were not statistically significant for some sensitivity analyses, including analyses stratified by hour of presentation and weekend (for Black patients) (eTable 6 in Supplement 1). Among patients who were not transferred from non–PCI-capable initial hospitals, Hispanic patients (but not Black patients) were more likely than White patients to receive fibrinolytics.

### Receipt of PCI Among Patients Initially Presenting to Non–PCI-Capable Hospitals and Transferred

**Figure 4.**
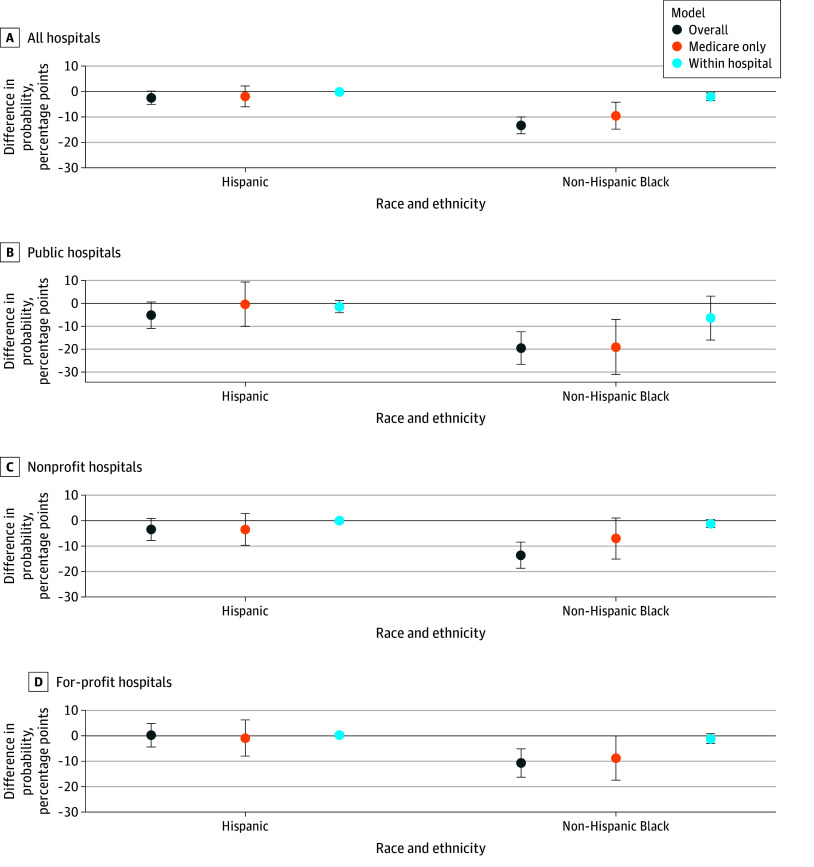
Probability of Receipt of Percutaneous Coronary Intervention (PCI) at a Receiving Hospital When a Patient With ST-Segment Elevation Myocardial Infarction Initially Presented to a Non–PCI-Capable Hospital and Was Transferred Non-Hispanic White patients were the comparison group. *Overall* indicates the overall population-based model, which used robust SEs and controlled for visit and hospital characteristics. *Medicare only* indicates the overall regression model in which the sample consisted only of Medicare patients. *Within hospital* indicates a hospital fixed-effects model that controlled for visit characteristics and hospital fixed effects. Other or missing race was excluded from the figure for clarity but included in the regression. Error bars indicate 95% CI.

When transferred from a non–PCI-capable hospital, 65.4% of White patients received PCI at the receiving hospital. Compared with White patients, Black patients were 20.3% less likely (–13.3 [95% CI, −16.6 to –9.9] pp; *P* < .001) to receive PCI at the receiving hospital (Figure 4). The effect sizes were smaller for Medicare patients and after controlling for differences within the same hospital. When stratified by ownership of the receiving hospital, Black patients were less likely than White patients to receive PCI at the receiving hospital. However, the differences were not statistically significant when restricting the sample to Medicare patients within nonprofit hospitals or after controlling for within-hospital differences. The differences between Hispanic and White patients were not statistically significant.

In most sensitivity analyses, Black patients were less likely than White patients to receive PCI at the receiving hospital (eTable 7 in Supplement 1). Compared with White patients who did not receive a PCI at the receiving hospital, Black patients were less likely to receive CABG and Hispanic patients were less likely to receive fibrinolytics (eTable 5 in Supplement 1). Across all outcomes, the E-value ranged from 1.3 to 2.8, suggesting that an unmeasured confounder would need to be associated with both the independent and dependent variables by a risk ratio of at least 1.5 to 2.8.

## Discussion

Our study estimated the extent to which stages in the patient care process contribute to racial and ethnic disparities in PCI and found that Black and Hispanic patients were less likely to receive PCI at every step of the process. Compared with White patients, Hispanic patients were 3.1 pp (3.8%) and Black patients were 1.8 pp (2.2%) less likely to present to a hospital that was PCI capable. Among patients initially presenting to PCI-capable hospitals, Black patients were 8.6 pp (10.7%) less likely to receive a PCI than White patients. Among patients with STEMI initially presenting to a non–PCI-capable hospital, Hispanic patients were 4.2 pp (5.6%) and Black patients were 4.0 pp (5.3%) less likely to be transferred to another hospital compared with White patients. Even if transferred, Black patients were 13.3 pp (20.3%) less likely to receive PCI at receiving hospitals compared with White patients.

These results are consistent with those of the 2007 study by Popescu et al,^9^ which found that Black Medicare patients with AMI were less likely than White patients to receive coronary revascularization and less likely to be transferred if they initially presented at a hospital without these capabilities. Our study extended their findings by analyzing an all-payer dataset and focusing specifically on STEMI care, for which clinical recommendations are clearer; because of this, it might be expected that disparities would be smaller.

Our study suggests that 15 years after the study by Popescu et al,^9^ disparities in STEMI care still exist. Our findings suggest that policies spanning the whole patient care process may be needed to reduce racial and ethnic disparities in receipt of PCI and should be rooted in a population health perspective that ultimately requires aligning incentives of stakeholders and patients.

### Proposed Policies to Decrease Disparities

Because most patients presented to a PCI-capable hospital (process A), the largest contributor to overall disparities in PCI use was the probability of PCI when the initial hospital was PCI capable (process B). Black patients with STEMI were 8.6 pp (10.7%) less likely to receive a PCI compared with White patients. Because Black patients were less likely than White patients to receive a PCI regardless of the hour at presentation or day of the week, availability of PCI was an unlikely explanation, as was payer, since disparities persisted among Medicare patients. Furthermore, because Black patients who did not receive PCI were not significantly more likely to use alternative therapies than White patients (eTable 5 in Supplement 1), delays in presentation after symptom onset, longer delays in diagnosis, and patient refusal likely were not explanations. The disparity was present even for patients within the same hospital and when stratified by PCI volume, suggesting that it was not explained by Black patients presenting to lower-quality or lower–PCI volume hospitals.^16,17,18,19^ The disparity may be because of clinician bias, such as erroneous assumptions about patient preferences, payer, or severity of symptoms at triage.^47^ This is consistent with prior studies suggesting that physicians are less likely to refer Black patients for intervention than White patients.^48^

Standardizing hospital protocols at PCI-capable hospitals to ensure eligible patients with STEMI receive PCI would improve equity since hospital protocols may reduce potential implicit bias. Hospital protocols should address differences in symptom presentation.^49^ In addition to hospital administrators supporting the adoption of protocols, government can also support their adoption. State-required adoption of hospital protocols for other conditions, such as sepsis, is associated with reductions in risk-adjusted mortality.^50,51^

The largest disparity in terms of magnitude was PCI use for patients who initially presented at a non–PCI-capable hospital and were transferred (process D). Black patients were 13.3 pp (20.3%) less likely to receive a PCI at the receiving hospital compared with White patients. However, this disparity was smaller within the same receiving hospital. This suggests that the disparity in receipt of PCI at receiving hospitals may be partially explained by initial hospitals being more likely to transfer Black patients, compared with White patients, to hospitals that are less likely to perform PCI. This finding may reflect differences in the ability of initial hospitals to find willing receiving hospitals. It may help explain prior research suggesting that Black patients may be initially transported or transferred to different hospitals than White patients.^21,52,53^

Insurance may explain transfer difficulty; the disparity in Black patients and White patients in transfer (4.0 pp [5.3%]) (process C) disappeared for Medicare patients only. Perhaps differences in transfer arise because initial hospitals have difficulty transferring uninsured and Medicaid patients because receiving hospitals are reluctant to accept them^54,55^ and Black patients are more likely to have Medicaid or no insurance than White patients.

If transfer difficulties are explained by insurance, policymakers may strengthen laws around transfers. These laws may include the Emergency Medical Treatment and Labor Act (EMTALA) and state analogues. Although EMTALA requires Medicare-participating hospitals with specialized capabilities to accept patients with emergency medical conditions if they have capacity and capability, admission decisions may be made by physicians who are unaware of EMTALA; thus, receiving hospitals may inappropriately deny these transfers.^54^ State policymakers can strengthen state analogues to EMTALA.

In addition, hospital policymakers can improve interhospital transfer agreements.^23^ Standardizing and formalizing these transfers may make it more likely that hospitals would transfer patients to hospitals best equipped to perform PCI.

The aforementioned policies should specifically target reducing disparities. Facially neutral policies targeted at improving STEMI care do not benefit all patients equally. For instance, communities with higher proportions of racial and ethnic minority individuals benefited least from regionalization.^12^ These policies should therefore explicitly determine whether they successfully reduced racial and ethnic disparities.

### Limitations

This study has limitations. First, because of data limitations, we used diagnoses at the initial hospital to determine the study cohort. For admitted patients (85.71%) (Table), these were hospital discharge diagnoses; for those not admitted, these were ED diagnoses, which may have resulted in some error. Second, the study may be limited in generalizability since we only examined 1 state. Nonetheless, the study results are important, as Florida is the third most populous US state. Future studies can examine other states. Third, the study is limited to available data. Clinical severity data, whether patients arrived by ambulance, and information about when symptoms started were missing. The E-values suggest that unmeasured confounding may help explain some results, but the likelihood varies by analysis (eTable 8 in Supplement 1).

## Conclusions

In this cross-sectional study, overall racial and ethnic disparities in PCI, particularly for Black patients with STEMI, reflected an accumulation of disparities manifesting across the care process and multiple hospitals. Our study suggests that 2 areas of focus could be standardizing hospital protocols at PCI-capable hospitals and improving hospital transfers.

## Notes

Supplement 1. **eFigure 1.** Overview of Study **eTable 1.** Diagnosis and Procedure Codes **eFigure 2.** ED Visits for STEMI **eTable 2.** Summary of Analysis, Sample, and Level of Hospital Characteristics **eTable 3.** Regression Results for Main Analysis **eTable 4.** Additional Analyses: PCI Utilization if Initially Present at PCI-Capable Hospital **eTable 5.** Alternative Therapies **eTable 6.** Additional Analyses: Transfer if Initially Present at a Non–PCI-Capable Hospital **eTable 7.** Additional Analyses: PCI Utilization at Receiving Hospital if Initially Present at a Non–PCI-Capable Hospital and Then Transferred **eTable 8.** E Values

Supplement 2.
Data Sharing Statement
