# Supplementary material for: Disparities by Race and Ethnicity in Percutaneous Coronary Intervention
Source: JAMA Netw Open. 2025 Sep 18;8(9):e2532660. doi: 10.1001/jamanetworkopen.2025.32660 (PMC12447256; doi:10.1001/jamanetworkopen.2025.32660)
Supplement: Supplement 1. — eFigure 1. Overview of Study eTable 1. Diagnosis and Procedure Codes eFigure 2. ED Visits for STEMI eTable 2. Summary of Analysis, Sample, and Level of Hospital Characteristics eTable 3. Regression Results for Main Analysis eTable 4. Additional Analyses: PCI Utilization if Initially Present at PCI-Capable Hospital eTable 5. Alternative Therapies eTable 6. Additional Analyses: Transfer if Initially Present at a Non–PCI-Capable Hospital eTable 7. Additional Analyses: PCI Utilization at Receiving Hospital if Initially Present at a Non–PCI-Capable Hospital and Then Transferred eTable 8. E Values [file jamanetwopen-e2532660-s001.pdf]

Supplementary Online Content

Hsuan C, Lin MP, Zebrowski A, et al. Disparities by race and ethnicity in percutaneous coronary intervention. *JAMA Netw Open.* 2025;8(9):e2532660. doi:10.1001/jamanetworkopen.2025.32660

eFigure 1. Overview of Study

eTable 1. Diagnosis and Procedure Codes

eFigure 2. ED Visits for STEMI

eTable 2. Summary of Analysis, Sample, and Level of Hospital Characteristics

eTable 3. Regression Results for Main Analysis

eTable 4. Additional Analyses: PCI Utilization if Initially Present at PCI-Capable Hospital

eTable 5. Alternative Therapies

eTable 6. Additional Analyses: Transfer if Initially Present at a Non–PCI-Capable Hospital

eTable 7. Additional Analyses: PCI Utilization at Receiving Hospital if Initially Present at a Non–PCI-Capable Hospital and Then Transferred

eTable 8. E Values

This supplementary material has been provided by the authors to give readers additional information about their work.

© 2025 Hsuan C et al. *JAMA Network Open*.

eFigure 1. Overview of Study

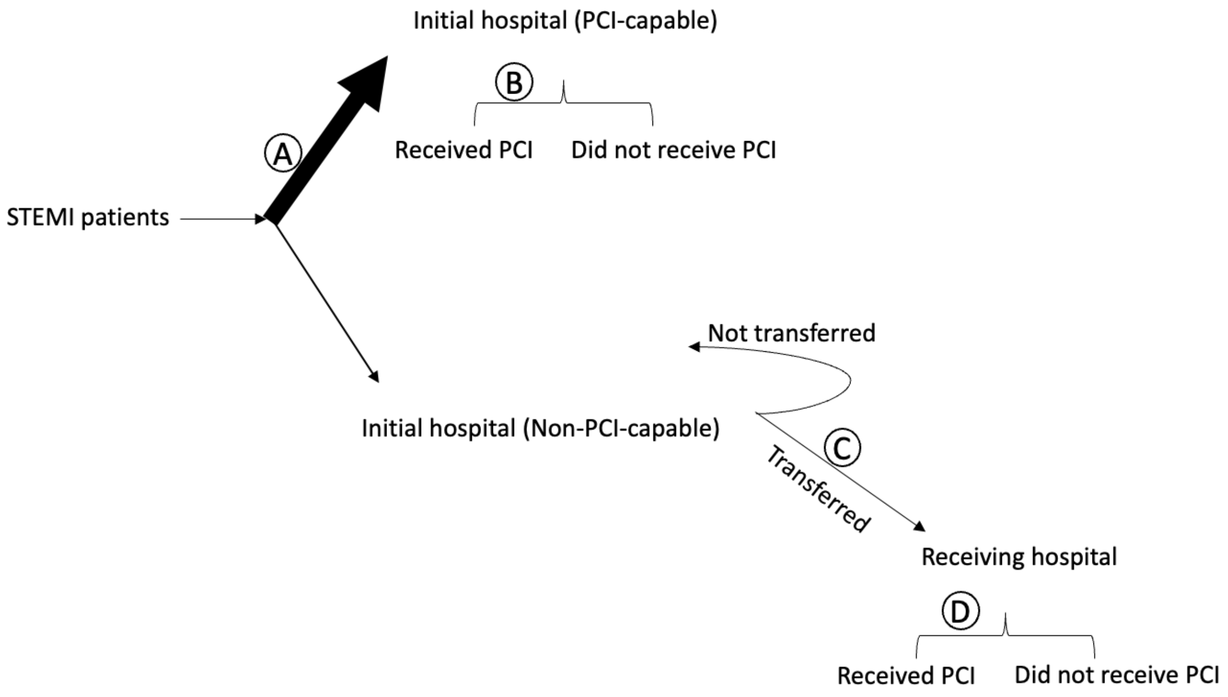

© 2025 Hsuan C et al. *JAMA Network Open*.

eTable 1. Diagnosis and Procedure Codes

	ICD9 diagnosis or procedure codes	ICD10 diagnosis or procedure codes	Source
STEMI	410.* except for 410.7*	I21.0* I21.1* I21.2* I21.3* I21.9* I22.0* I22.1* I22.8* I22.9*	Carr BG, Kilaru AS, Karp DN, Delgado MK, Wiebe DJ. Quality Through Coopetition: An Empiric Approach to Measure Population Outcomes for Emergency Care-Sensitive Conditions. Ann Emerg Med. 2018 Sep;72(3):237-245.
PCI	0066 3601 3602 3605 3606 3607 3609	0270346 027034Z 0270356 027035Z 0270366 027036Z 0270376 027037Z 02703D6 02703DZ 02703E6 02723E6 02723EZ 02723F6 02723FZ 02723G6 02723GZ 02723Z6 02723ZZ 0272446 027244Z 0272456 02703EZ 02703F6 02703FZ 02703G6 02703GZ 02703Z6 02703ZZ 0270446 027044Z 0270456 027045Z 027245Z 0272466 027246Z 0272476 027247Z 02724D6 02724DZ 02724E6 02724EZ 02724F6 02724FZ 0270466 027046Z 0270476 027047Z 02704D6 02704DZ 02704E6 02704EZ 02704F6 02704FZ 02704G6 02724G6 02724GZ 02724Z6 02724ZZ 0273346 027334Z 0273356 027335Z 0273366 02704GZ 02704Z6 02704ZZ 0271346 027134Z 0271356 027135Z 0271366 027136Z 0271376 027137Z 02713D6 027337Z 02733D6 02733DZ 02733E6 02733EZ 02733F6 02733FZ 02733G6 02733GZ 02733Z6 02733ZZ 0273446 02713DZ 02713E6 02713EZ 02713F6 02713FZ 02713G6 02713GZ 02713Z6 02713ZZ 0271446 027144Z 027344Z 0273456 027345Z 0273466 027346Z 0273476 027347Z 02734D6 02734DZ 02734E6 02734EZ 0271456 027145Z 0271466 027146Z 0271476 027147Z 02714D6 02714DZ 02714E6 02714EZ 02714F6 02714FZ 02734F6 02734FZ 02734G6 02734GZ 02734Z6 02734ZZ 02C03Z6 02C03Z7 02C03ZZ 02C13Z6 02C13Z7 02C13ZZ 02714G6 02714GZ 02714Z6 02714ZZ 0272346 027234Z 0272356 027235Z 0272366 027236Z 0272376 027237Z 02C23Z6 02C23Z7 02C23ZZ 02C33Z6 02C33Z7 02C33ZZ 02H03DZ 02H13DZ 02H23DZ 02H33DZ X2C0361 X2C1361 02723D6 02723DZ X2C2361 X2C3361	Agency for Healthcare Research and Quality. (2024) Inpatient Quality Indicator 06 (IQI 06) Percutaneous Coronary Intervention (PCI) Volume. Available at:<br><br>Concannon TW, Nelson J, Goetz J, Griffith JL. A percutaneous coronary intervention lab in every hospital? Circ Cardiovasc Qual Outcomes. 2012 Jan;5(1):14-20

© 2025 Hsuan C et al. *JAMA Network Open*.

Fibrinolytics	V45.88; 99.10	Z92.82; 3E04317; 3E03317	Elbadawi A, Mahtta D, Elgendy IY, Saad M, Krittanawong C, Hira RS, Omer M, Ogunbayo GO, Garratt K, Rao SV, Jneid H. Trends and Outcomes of Fibrinolytic Therapy for STEMI: Insights and Reflections in the COVID-19 Era. JACC Cardiovasc Interv. 2020 Oct 12;13(19):2312-2314.
CABG	3610 3611 3612 3613 3614 3615 3616 3617 3619	0210083 0210088 0210089 021008C 021008F 021008W 0210093 0210098 0210099 021009C 021009F 021009W 0212083 0212088 0212089 021208C 021208F 021208W 0212093 0212098 0212099 021209C 021209F 021209W 02100A3 02100A8 02100A9 02100AC 02100AF 02100AW 02100J3 02100J8 02100J9 02100JC 02100JF 02100JW 02120A3 02120A8 02120A9 02120AC 02120AF 02120AW 02120J3 02120J8 02120J9 02120JC 02120JF 02120JW 02100K3 02100K8 02100K9 02100KC 02100KF 02100KW 02100Z3 02100Z8 02100Z9 02100ZC 02100ZF 0211083 02120K3 02120K8 02120K9 02120KC 02120KF 02120KW 02120Z3 02120Z8 02120Z9 02120ZC 02120ZF 0213083 0211088 0211089 021108C 021108F 021108W 0211093 0211098 0211099 021109C 021109F 021109W 0213088 0213089 021308C 021308F 021308W 0213093 0213098 0213099 021309C 021309F 021309W 02110A3 02110A8 02110A9 02110AC 02110AF 02110AW 02110J3 02110J8 02110J9 02110JC 02110JF 02130A3 02130A8 02130A9 02130AC 02130AF 02130AW 02130J3 02130J8 02130J9 02130JC 02130JF 02110JW 02110K3 02110K8 02110K9 02110KC 02110KF 02110KW 02110Z3 02110Z8 02110Z9 02110ZC 02110ZF 02130JW 02130K3 02130K8 02130K9 02130KC 02130KF 02130KW 02130Z3 02130Z8 02130Z9 02130ZC 02130ZF	Agency for Healthcare Research and Quality. (2024) Inpatient Quality Indicator 12 (IQI 12) Coronary Artery Bypass Graft (CABG) Mortality Rate. Available at: https://qualityindicators.ahrq.gov/Downloads/Modules/IQI/V60/TechSpecs/IQI_12_Coronary_Artery_Bypass_Graft_(CABG)_Mortality_Rate.pdf

© 2025 Hsuan C et al. *JAMA Network Open*.

eFigure 2. ED Visits for STEMI

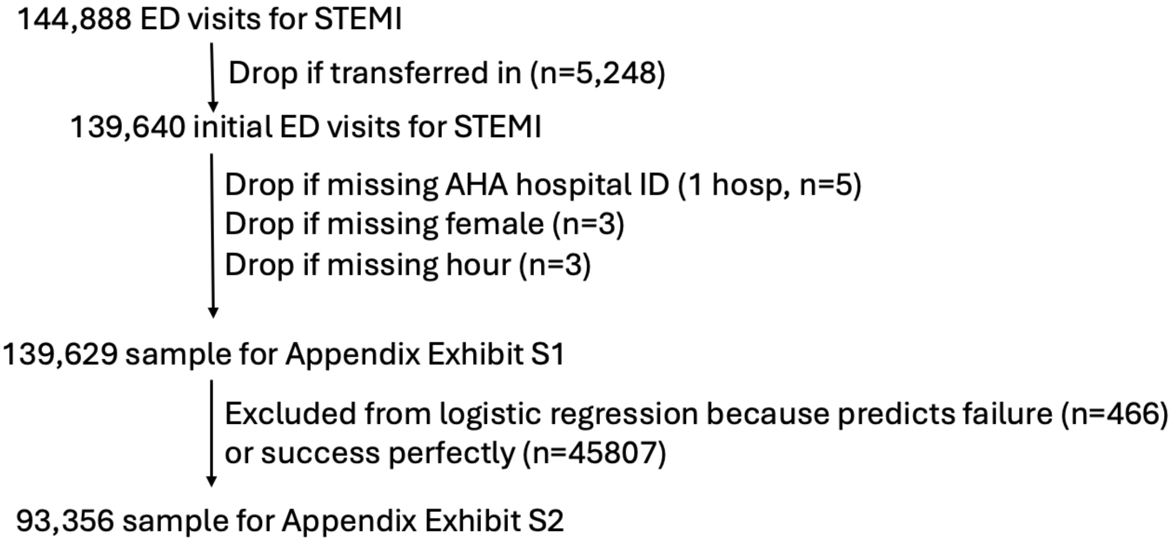

© 2025 Hsuan C et al. *JAMA Network Open*.

eTable 2. Summary of Analysis, Sample, and Level of Hospital Characteristics

Path	Analyses	Sample	Hospital characteristics / fixed effects
Presentation to PCI-capable hospital (Process A)	1. Overall<br>2. Medicare only	STEMI patients	Initial hospital
If initially present to PCI-capable hospital, receipt of PCI at initial hospital (Process B)	1. Overall<br>2. Medicare only<br>3. Hospital fixed effects<br>4. Stratified by hospital ownership	STEMI patients who initially presented to PCI capable hospital<br><br>Excludes patients who died in the ED or who were transferred	Initial hospital
If initially present to non-PCI-capable hospital, transfer to another hospital (Process C)	1. Overall<br>2. Medicare only<br>3. Hospital fixed effects	STEMI patients who initially presented to non-PCI-capable hospital<br><br>Excludes patients who died in the ED or who received a PCI at the initial hospital	Initial hospital
If initially present to non-PCI-capable hospital and transferred, receipt of PCI at receiving hospital (Process D)	1. Overall<br>2. Medicare only<br>3. Hospital fixed effects<br>4. Stratified by hospital ownership	STEMI patients who initially presented to non-PCI-capable hospital and were transferred<br><br>Excludes patients who died in the ED of the receiving hospital or who were transferred again	Receiving hospital

© 2025 Hsuan C et al. *JAMA Network Open*.

eTable 3. Regression Results for Main Analysis

A. Initial Presentation to PCI-Capable Hospital

	Overall OR (SE)	Medicare Only OR (SE)
Hispanic	0.701***	0.642***
	(0.027)	(0.037)
Black non-Hispanic	0.805***	0.715***
	(0.035)	(0.049)
White non-Hispanic	[ref]	[ref]
Other/Missing Race	1.084	0.948
	(0.068)	(0.100)
self	0.907**	
	(0.034)	
Medicaid	0.836***	
	(0.041)	
Medicare	0.922*	
	(0.033)	
Private	[ref]	
Other/missing	1.057	
	(0.071)	
N	93,356	47,379

B. Receipt of PCI if Initially Present to PCI-Capable Hospital

1. All Hospitals

	Overall OR (SE)	Medicare OR (SE)	Hosp FE OR (SE)
Hispanic	0.960	0.939	0.934*
	(0.025)	(0.034)	(0.026)
Black non-Hispanic	0.596***	0.622***	0.606***
	(0.016)	(0.024)	(0.016)
White non-Hispanic	[ref]	[ref]	[ref]
Other/Missing Race	0.993	1.059	1.007
	(0.035)	(0.057)	(0.036)
self	0.934*		0.950
	(0.027)		(0.028)
Medicaid	0.734***		0.745***

© 2025 Hsuan C et al. *JAMA Network Open*.

	(0.025)		(0.025)
Medicare	0.861***		0.860***
	(0.022)		(0.021)
Private	[ref]		[ref]
Other/missing	0.700***		0.683***
	(0.030)		(0.030)
N	116,554	57,578	116,549

2. Public Hospitals Only

	Overall<br>OR (SE)	Medicare<br>OR (SE)	Hosp FE<br>OR (SE)
Hispanic	1.038	0.965	1.039
	(0.067)	(0.089)	(0.067)
Black non-Hispanic	0.724***	0.747**	0.714***
	(0.047)	(0.073)	(0.046)
White non-Hispanic	[ref]	[ref]	[ref]
Other/Missing Race	0.879	0.899	0.869
	(0.101)	(0.154)	(0.102)
self	0.970		0.972
	(0.071)		(0.072)
Medicaid	0.806**		0.809*
	(0.067)		(0.067)
Medicare	0.872*		0.873*
	(0.058)		(0.057)
Private	[ref]		[ref]
Other/missing	0.699**		0.695**
	(0.085)		(0.085)
N	17,316	8,226	17,316

3. Non-profit hospitals

	Overall<br>OR (SE)	Medicare<br>OR (SE)	Hosp FE<br>OR (SE)
Hispanic	0.896**	0.864**	0.917*
	(0.035)	(0.048)	(0.036)
Black non-Hispanic	0.630***	0.671***	0.620***
	(0.024)	(0.037)	(0.024)
White non-Hispanic	[ref]	[ref]	[ref]
Other/Missing Race	0.972	0.994	0.982

© 2025 Hsuan C et al. *JAMA Network Open*.

	(0.048)	(0.073)	(0.049)
Self	0.974		0.971
	(0.042)		(0.042)
Medicaid	0.729***		0.720***
	(0.037)		(0.036)
Medicare	0.876***		0.869***
	(0.032)		(0.031)
Private	[ref]		[ref]
Other/missing	0.750***		0.740***
	(0.046)		(0.046)
N	55,829	27,389	55,824

4. For profit hospitals

	Overall OR (SE)	Medicare OR (SE)	Hosp FE OR (SE)
Hispanic	0.940	0.967	0.916
	(0.041)	(0.058)	(0.044)
Black non-Hispanic	0.528***	0.557***	0.543***
	(0.023)	(0.035)	(0.024)
White non-Hispanic	[ref]	[ref]	[ref]
Other/Missing Race	1.034	1.188	1.076
	(0.060)	(0.108)	(0.063)
self	0.902*		0.920
	(0.042)		(0.043)
Medicaid	0.741***		0.742***
	(0.041)		(0.041)
Medicare	0.847***		0.846***
	(0.035)		(0.035)
Private	[ref]		[ref]
Other/missing	0.628***		0.615***
	(0.043)		(0.043)
N	43,409	21,963	43,409

C. Transfer if Initially Present to non-PCI-capable hospital

	Overall OR (SE)	Medicare OR (SE)	Hosp FE OR (SE)
Hispanic	0.787***	1.165	0.728***
	(0.049)	(0.120)	(0.048)

© 2025 Hsuan C et al. *JAMA Network Open*.

Black non-Hispanic	0.795***	1.029	0.820**
	(0.055)	(0.117)	(0.058)
White non-Hispanic	[ref]	[ref]	[ref]
Other/Missing Race	0.677***	0.985	0.620***
	(0.073)	(0.183)	(0.068)
self	0.778***		0.781***
	(0.047)		(0.049)
Medicaid	1.051		1.064
	(0.084)		(0.086)
Medicare	1.839***		1.856***
	(0.117)		(0.119)
Private	[ref]		[ref]
Other/missing	0.970		0.962
	(0.119)		(0.117)
N	15,932	7,658	15,911

D. Receipt of PCI at Receiving Hospital, if Initially Presented to non-PCI-capable Hospital and then Transferred

1. All Hospitals

	Overall<br>OR (SE)	Medicare<br>OR (SE)	Hosp FE<br>OR (SE)
Hispanic	0.891	0.920	0.924
	(0.059)	(0.087)	(0.066)
Black non-Hispanic	0.542***	0.655***	0.531***
	(0.042)	(0.078)	(0.041)
White non-Hispanic	[ref]	[ref]	[ref]
Other/Missing Race	0.995	0.858	0.984
	(0.132)	(0.174)	(0.131)
self	0.841*		0.866*
	(0.059)		(0.061)
Medicaid	0.669***		0.674***
	(0.058)		(0.059)
Medicare	0.662***		0.675***
	(0.044)		(0.045)
Private	[ref]		[ref]
Other/missing	0.793		0.797
	(0.102)		(0.106)
N	11,067	5,160	11,033

© 2025 Hsuan C et al. *JAMA Network Open*.

2. Public Hospitals

	Overall OR (SE)	Medicare OR (SE)	Hosp FE OR (SE)
Hispanic	0.769	0.983	0.771
	(0.114)	(0.231)	(0.113)
Black non-Hispanic	0.397***	0.422**	0.397***
	(0.067)	(0.117)	(0.066)
White non-Hispanic	[ref]	[ref]	[ref]
Other/Missing Race	0.875	0.909	0.878
	(0.254)	(0.390)	(0.252)
self	0.908		0.906
	(0.146)		(0.147)
Medicaid	0.638*		0.641*
	(0.131)		(0.132)
Medicare	0.643**		0.642**
	(0.105)		(0.104)
Private	[ref]		[ref]
Other/missing	0.968		0.960
	(0.326)		(0.321)
N	1,967	830	1,967

3. Non-profit Hospitals

	Overall OR (SE)	Medicare OR (SE)	Hosp FE OR (SE)
Hispanic	0.842	0.845	0.892
	(0.089)	(0.128)	(0.097)
Black non-Hispanic	0.523***	0.721	0.541***
	(0.064)	(0.138)	(0.065)
White non-Hispanic	[ref]	[ref]	[ref]
Other/Missing Race	1.021	0.684	1.023
	(0.217)	(0.225)	(0.217)
self	0.863		0.859
	(0.091)		(0.091)
Medicaid	0.637***		0.648**
	(0.086)		(0.087)
Medicare	0.771**		0.790*
	(0.077)		(0.078)
Private	[ref]		[ref]

© 2025 Hsuan C et al. *JAMA Network Open*.

Other/missing	0.924		0.931
	(0.175)		(0.181)
N	4,907	2,196	4,898

4. **For profit hospitals**

	Overall OR (SE)	Medicare OR (SE)	Hosp FE OR (SE)
Hispanic	1.008	0.960	1.125
	(0.114)	(0.155)	(0.143)
Black non-Hispanic	0.612***	0.677*	0.627***
	(0.078)	(0.131)	(0.083)
White non-Hispanic	[ref]	[ref]	[ref]
Other/Missing Race	1.018	1.033	0.997
	(0.229)	(0.352)	(0.215)
self	0.821		0.856
	(0.096)		(0.101)
Medicaid	0.705*		0.711*
	(0.100)		(0.101)
Medicare	0.565***		0.564***
	(0.062)		(0.062)
Private	[ref]		[ref]
Other/missing	0.589*		0.584*
	(0.130)		(0.129)
N	4,180	2,117	4,159

Source/Notes: Authors' analysis of data.
NOTES: Logistic regression examining differences in race/ethnicity on receipt of PCI when a patient initially presents to a PCI-capable hospital, both in all hospitals and as stratified by hospital ownership. A PCI-capable hospital is defined as a hospital that in the prior year performed at least 50 PCIs. "Overall" indicates the overall, population-based model, which uses robust standard errors and controls for visit characteristics (payer, age, age-squared, sex, day of week, hour, Charlson comorbidity index, patient home distance from hospital, and year) and hospital characteristics (bed size, academic status, ownership, hospital referral region, and urbanicity). "Medicare only" indicates the overall regression model where the sample consists only of Medicare patients. "Within Hospital" indicates a hospital fixed effects model, which controls for visit characteristics and hospital fixed effects, and indicates the difference as compared to a White non-Hispanic patient within the same hospital.
Hosp FE = hospital fixed effects; OR = odds ratio; SE = standard error.

© 2025 Hsuan C et al. *JAMA Network Open*.

eTable 4. Additional Analyses: PCI Utilization if Initially Present at PCI-Capable Hospital

a. By Hour of ED Registration

We stratified analyses by the hour of ED registration (12-7 AM, 8 AM-3 PM, 4-11 PM) and whether patients presented on a weekday or weekend.

	12-7 AM<br>OR (SE)	8 AM-3 PM<br>OR (SE)	4-11 PM<br>OR (SE)
Hispanic	0.894	0.969	0.959
	(0.056)	(0.040)	(0.047)
Black non-Hispanic	0.612***	0.571***	0.629***
	(0.036)	(0.024)	(0.030)
White non-Hispanic	[ref]	[ref]	[ref]
Other/Missing Race	0.983	1.093	0.949
	(0.088)	(0.063)	(0.061)
self	0.995	0.913*	1.030
	(0.065)	(0.042)	(0.055)
Medicaid	0.836*	0.762***	0.738***
	(0.066)	(0.042)	(0.045)
Medicare	0.882*	0.871***	0.879**
	(0.052)	(0.035)	(0.041)
Private	[ref]	[ref]	[ref]
Other/missing	0.785*	0.629***	0.778**
	(0.078)	(0.041)	(0.063)
N	23,025	49,197	34,990

b. By weekend/weekday

	Weekday<br>OR (SE)	Weekend<br>OR (SE)
Hispanic	0.933*	0.997
	(0.031)	(0.054)
Black non-Hispanic	0.588***	0.631***
	(0.020)	(0.033)
White non-Hispanic	[ref]	[ref]
Other/Missing Race	1.043	0.962
	(0.048)	(0.069)
self	0.979	0.949

© 2025 Hsuan C et al. *JAMA Network Open*.

	(0.036)	(0.054)
Medicaid	0.770***	0.767***
	(0.033)	(0.053)
Medicare	0.885***	0.855**
	(0.028)	(0.044)
Private	[ref]	[ref]
Other/missing	0.759***	0.594***
	(0.041)	(0.049)
N	76,388	30,824

c. PCI Volume (% of STEMI patients who received PCI, by quartiles)

Since racial and ethnic minorities are more likely to go to hospitals that perform fewer PCIs, and hospitals that perform fewer PCIs in general might be less likely to perform PCIs on a specific patient, we stratified by PCI volume the prior year. Specifically, we calculated the percent of STEMI patients who received a PCI at that hospital the prior year, and then calculated the quartile of PCIs conducted. Hospitals in quartile 1 performed the fewest PCIs the previous year; hospitals in quartile 4 performed the most PCIs the previous year.

	Q1<br>OR (SE)	Q2<br>OR (SE)	Q3<br>OR (SE)	Q4<br>OR (SE)
Hispanic	0.903	0.927	0.942	0.985
	(0.050)	(0.051)	(0.053)	(0.059)
Black non-Hispanic	0.608***	0.654***	0.612***	0.574***
	(0.036)	(0.037)	(0.033)	(0.033)
White non-Hispanic	[ref]	[ref]	[ref]	[ref]
Other/Missing Race	1.000	1.029	1.021	1.051
	(0.084)	(0.077)	(0.074)	(0.087)
self	1.055	0.941	0.865*	1.094
	(0.066)	(0.059)	(0.052)	(0.068)
Medicaid	0.845*	0.752***	0.740***	0.787***
	(0.063)	(0.056)	(0.052)	(0.056)
Medicare	0.989	0.801***	0.820***	0.935
	(0.055)	(0.043)	(0.043)	(0.051)
Private	[ref]	[ref]	[ref]	[ref]
Other/missing	0.806*	0.603***	0.680***	0.722***
	(0.084)	(0.053)	(0.059)	(0.064)
N	21,236	25,688	28,875	31,372

© 2025 Hsuan C et al. *JAMA Network Open*.

d. Excluding 2020-2021 and using ICD9 and ICD10 years only

We excluded 2020-2021 from the analysis in case the COVID-19 pandemic, which disrupted ED use, changed PCI utilization (e.g. if Black non-Hispanic patients were more likely to delay going to the ED compared with White non-Hispanic patients). We also stratified analyses by whether the patient diagnoses were coded under ICD-9 or ICD-10 diagnosis codes.

	ICD9<br>OR (SE)	ICD10<br>OR (SE)	No 2020-21<br>OR (SE)
Hispanic	0.978	0.943	0.968
	(0.046)	(0.034)	(0.031)
Black non-Hispanic	0.622***	0.586***	0.614***
	(0.028)	(0.021)	(0.019)
White non-Hispanic	[ref]	[ref]	[ref]
Other/Missing Race	1.055	1.007	1.067
	(0.070)	(0.048)	(0.048)
self	0.904*	0.991	0.926*
	(0.044)	(0.039)	(0.032)
Medicaid	0.697***	0.807***	0.741***
	(0.039)	(0.038)	(0.030)
Medicare	0.882**	0.860***	0.861***
	(0.038)	(0.030)	(0.026)
Private	[ref]	[ref]	[ref]
Other/missing	0.621***	0.736***	0.676***
	(0.045)	(0.043)	(0.035)
Observations	43,781	63,431	87,265

e. Including patients who died/transferred, excluding patients who left against medical advice, excluding patients who had diabetes, or including males only

	Died/Tran<br>OR (SE)	Against Medical Advice<br>OR (SE)	No Diabetes<br>OR (SE)	Men only<br>OR (SE)
Hispanic	0.971	0.949	0.950	0.983
	(0.024)	(0.027)	(0.027)	(0.034)
Black non-Hispanic	0.585***	0.609***	0.600***	0.555***
	(0.014)	(0.017)	(0.017)	(0.020)
White non-Hispanic	[ref]	[ref]	[ref]	[ref]

© 2025 Hsuan C et al. *JAMA Network Open*.

Other/Missing Race	0.988	1.010	1.020	1.042
	(0.033)	(0.040)	(0.040)	(0.049)
self	0.890***	1.007	0.968	0.895**
	(0.023)	(0.032)	(0.030)	(0.032)
Medicaid	0.730***	0.782***	0.767***	0.715***
	(0.023)	(0.029)	(0.028)	(0.032)
Medicare	0.845***	0.889***	0.874***	0.827***
	(0.020)	(0.024)	(0.024)	(0.027)
Private	[ref]	[ref]	[ref]	[ref]
Other/missing	0.698***	0.718***	0.705***	0.726***
	(0.028)	(0.033)	(0.032)	(0.037)
N	122,387	105,365	107,212	74,991

f. Urbanicity

	Large Central Metro	Large Fringe Metro	Medium Metro	Small metro, micropolitan & non-core
Hispanic	OR (SE)	OR (SE)	OR (SE)	OR (SE)
	(0.040)	(0.048)	(0.058)	(0.133)
Non-Hispanic Black	0.652***	0.631***	0.541***	0.507***
	(0.029)	(0.036)	(0.027)	(0.069)
Non-Hispanic White	[ref]	[ref]	[ref]	[ref]
Other/Missing Race & Ethn	0.978	1.004	1.113	0.876
	(0.059)	(0.074)	(0.088)	(0.143)
self	0.971	0.923	1.039	0.945
	(0.048)	(0.056)	(0.059)	(0.114)
Medicaid	0.741***	0.778***	0.847*	0.806
	(0.042)	(0.058)	(0.058)	(0.120)
Medicare	0.816***	0.923	0.892*	0.973
	(0.038)	(0.049)	(0.042)	(0.104)
Private	[ref]	[ref]	[ref]	[ref]
Other/missing	0.646***	0.782*	0.784**	0.554***
	(0.048)	(0.079)	(0.062)	(0.080)
N	36,753	27,073	35,033	8,353

g. Size of hospital

	<200 bed	200-500	>500
	OR (SE)	OR (SE)	OR (SE)

© 2025 Hsuan C et al. *JAMA Network Open*.

Hispanic	0.824	0.959	0.975
	(0.085)	(0.040)	(0.042)
Black non-Hispanic	0.523***	0.551***	0.701***
	(0.049)	(0.022)	(0.031)
White non-Hispanic	[ref]	[ref]	[ref]
Other/Missing Race	0.849	1.082	1.003
	(0.113)	(0.059)	(0.062)
self	0.927	0.966	1.000
	(0.081)	(0.042)	(0.051)
Medicaid	0.730**	0.844**	0.710***
	(0.078)	(0.045)	(0.040)
Medicare	0.956	0.871***	0.852***
	(0.074)	(0.033)	(0.038)
Private	[ref]	[ref]	[ref]
Other/missing	0.759*	0.669***	0.729***
	(0.099)	(0.042)	(0.055)
N	14,433	56,546	36,233

h. Alternative definition of PCI-capable and hospital

In the main analysis, we used a threshold of 50 PCIs the prior year to define a hospital as PCI-capable. In sensitivity analyses, we used a threshold of 4 or more PCIs the prior year to define a hospital as PCI-capable.

In Florida, hospitals apply for a license for Adult Inpatient Diagnostic Cardiac Catheterization, which is subset into Level I and Level II programs that authorize adult PCI with and without onsite cardiac surgery, respectively. (1) Hospitals applying for this license must perform a threshold number of PCIs performed, which varies for Level I and Level II hospitals and by distance between Level I and II hospitals. In addition, they must meet the American College of Cardiology and the American Heart Association guidelines for staffing, physician training and experience, and have demonstration plans to provide services to Medicaid and charity care patients. The thresholds Florida used to determine whether a hospital was state-licensed as a Level I or Level II hospital was much higher than the definition of PCI-capable hospital in prior literature (300 for a Level I hospital). In sensitivity analyses, we defined a PCI-capable hospital as a hospital that was either a Level I or Level II hospital.

The main analysis defines hospital based on AHA hospital identifiers since we use AHA to define key variables including location. In the sensitivity analysis, we used the hospital identifier as defined by Florida.

	PCI4	State def	Alt hosp
	OR (SE)	OR (SE)	OR (SE)

© 2025 Hsuan C et al. *JAMA Network Open*.

Hispanic	0.958	0.875***	0.948
	(0.027)	(0.026)	(0.027)
Black non-Hispanic	0.601***	0.602***	0.594***
	(0.017)	(0.017)	(0.017)
White non-Hispanic	[ref]	[ref]	[ref]
Other/Missing Race	1.021	1.005	1.004
	(0.039)	(0.040)	(0.040)
self	0.964	0.951	1.012
	(0.029)	(0.030)	(0.032)
Medicaid	0.770***	0.802***	0.779***
	(0.028)	(0.030)	(0.029)
Medicare	0.875***	0.896***	0.860***
	(0.023)	(0.025)	(0.024)
Private	[ref]	[ref]	[ref]
Other/missing	0.704***	0.731***	0.698***
	(0.032)	(0.034)	(0.032)
Observations	108,096	98,771	106,146

Notes. FE = fixed effects; OR = odds ratio; SE = standard error. Model uses robust standard errors and controls for visit characteristics (payer, age, age-squared, sex, weekend/weekday, hour, Charlson comorbidity index, patient home distance from hospital, and year) and hospital characteristics (bed size, academic status, ownership, hospital referral region, and urbanicity).

© 2025 Hsuan C et al. *JAMA Network Open*.

eTable 5. Alternative Therapies

We examined whether patients who did not receive a PCI received fibrinolytics or coronary artery bypass graft (CABG) surgery.

Fibrinolytics is not preferred treatment of STEMI, as almost half of patients with fibrinolysis either do not achieve reperfusion or have ineffective reperfusion. (2) However, if there is long delay between symptom onset and treatment, then fibrinolytics may be warranted. (2) Fibrinolytics were determined using a combination of ICD9/ICD10 diagnosis and procedure codes. (Appendix I) Because Florida emergency department data after 2018 does not include ICD-10 procedure codes (although it is included in inpatient data), we included only before 2018 for this sample.

A higher odds ratio indicates that, compared with White patients, a Black non-Hispanic patient or a Hispanic patient was more likely to receive fibrinolytics.

We also examined CABG surgery. CABG is not usually recommended to treat patients with STEMI, as excess mortality is higher for CABG than for PCIs, but it can sometimes be indicated depending on coronary anatomy or if the patient has a coexisting vulvular heart disease or diabetes. (2) We include in sensitivity analyses instead of the main analysis because CABG may not performed at the index STEMI presentation, but rather at a later hospitalization, and since we only have hospital discharge data that do not have clinical detail, it would not be clear if a CABG conducted at a later hospitalization was for the STEMI or because of a later diagnosis for multi-vessel disease.

A higher odds ratio indicates that, compared with White patients, a Black non-Hispanic patient or a Hispanic patient was more likely to receive CABG.

Only three patients received CABG if they initially presented to a non-PCI-capable hospital, did not die and did not receive a PCI at the initial hospital, so we examine only fibrinolytics for that sample.

a. **Receive fibrinolytics at initial hospital? (before 2018 only)**

	Lytic OR (SE)	Lytic-Mcare OR (SE)	Lytic-Hosp OR (SE)
Hispanic	0.958	0.716	0.793
	(0.259)	(0.351)	(0.236)
Non-Hispanic Black	0.980	1.078	0.922
	(0.226)	(0.451)	(0.223)
Non-Hispanic White	[ref]	[ref]	[ref]
Other/Missing Race & Ethn	1.253	2.466	1.383
	(0.434)	(1.320)	(0.514)

© 2025 Hsuan C et al. *JAMA Network Open*.

self	1.170		1.035
	(0.263)		(0.251)
Medicaid	0.711		0.700
	(0.242)		(0.245)
Medicare	0.791		0.836
	(0.202)		(0.238)
Private	[ref]		[ref]
Other/missing	1.674		1.714
	(0.520)		(0.596)
Observations	12,211	6,859	7,983

b. **Receive CABG at initial hospital?**

	CABG OR (SE)	CABG-Mcare OR (SE)	CABG-Hosp OR (SE)
Hispanic	1.093	1.086	1.163*
	(0.069)	(0.099)	(0.081)
Black non-Hispanic	0.522***	0.527***	0.549***
	(0.038)	(0.060)	(0.041)
White non-Hispanic	[ref]	[ref]	[ref]
Other/Missing Race	1.238*	1.152	1.203*
	(0.108)	(0.159)	(0.106)
self	0.902		0.930
	(0.065)		(0.070)
Medicaid	0.821*		0.846
	(0.070)		(0.073)
Medicare	0.843**		0.826**
	(0.050)		(0.051)
Private	[ref]		[ref]
Other/missing	0.482***		0.483***
	(0.058)		(0.058)
Observations	19,574	11,563	16,710

c. **Receive fibrinolytics if initially present to a non-PCI-capable hospital and was not transferred?**

Note: hospital fixed effects model did not converge

	Lytic OR (SE)	Lytic-Mcare OR (SE)

© 2025 Hsuan C et al. *JAMA Network Open*.

Hispanic	2.135**	1.124
	(0.520)	(0.637)
Black non-Hispanic	1.156	1.168
	(0.319)	(0.664)
White non-Hispanic	[ref]	[ref]
Other/Missing Race	1.110	1.000
	(0.505)	
Self	0.840	
	(0.176)	
Medicaid	0.651	
	(0.212)	
Medicare	0.589	
	(0.160)	
Private	[ref]	
Other/missing	0.772	
	(0.387)	
Observations	2,909	1,466

d. Receive fibrinolytics at receiving hospital, if initially present to a non-PCI-capable hospital and was transferred?

	Lytic OR (SE)	Lytic-Mcare OR (SE)	Lytic-Hosp OR (SE)
Hispanic	0.300**	0.339*	0.344*
	(0.126)	(0.162)	(0.143)
Non-Hispanic Black	0.497	0.603	0.533
	(0.207)	(0.267)	(0.227)
Non-Hispanic White	[ref]	[ref]	[ref]
Other/Missing Race & Ethn	1.511	1.861	1.653
	(0.965)	(1.269)	(1.002)
Self	1.383		1.508
	(0.473)		(0.534)
Medicaid	0.327		0.348
	(0.221)		(0.226)
Medicare	0.659		0.709
	(0.239)		(0.283)
Private	[ref]		[ref]
Other/missing	0.886		0.953

© 2025 Hsuan C et al. *JAMA Network Open*.

	(0.682)		(0.755)
Observations	2,690	1,944	1,822

e. **Receive CABG at receiving hospital, if initially present to a non-PCI-capable hospital and transferred?**

	CABG<br>OR (SE)	CABG-Mcare<br>OR (SE)	CABG-Hosp<br>OR (SE)
Hispanic	0.870	0.859	1.091
	(0.148)	(0.144)	(0.210)
Black non-Hispanic	0.420***	0.403***	0.467***
	(0.091)	(0.087)	(0.108)
White non-Hispanic	[ref]	[ref]	[ref]
Other/Missing Race	1.309	1.270	1.261
	(0.412)	(0.408)	(0.402)
self	1.113		1.140
	(0.196)		(0.208)
Medicaid	0.625*		0.591*
	(0.144)		(0.138)
Medicare	0.507***		0.535***
	(0.081)		(0.091)
Private	[ref]		[ref]
Other/missing	0.482		0.482*
	(0.183)		(0.175)
Observations	2,720	2,720	2,432
	~~0.870~~	~~0.859~~	~~1.091~~

Source/Notes: Authors' analysis of data.
NOTES: Logistic regression examining differences in race/ethnicity on receipt of PCI when a patient initially presents to a PCI-capable hospital, both in all hospitals and as stratified by hospital ownership. A PCI-capable hospital is defined as a hospital that in the prior year performed at least 50 PCIs. "Overall" indicates the overall, population-based model, which uses robust standard errors and controls for visit characteristics (payer, age, age-squared, sex, day of week, hour, Charlson comorbidity index, patient home distance from hospital, and year) and hospital characteristics (bed size, academic status, ownership, hospital referral region, and urbanicity). "Medicare only" indicates the overall regression model where the sample consists only of Medicare patients. "Within Hospital" indicates a hospital fixed effects model, which

© 2025 Hsuan C et al. *JAMA Network Open*.

controls for visit characteristics and hospital fixed effects, and indicates the difference as compared to a White non-Hispanic patient within the same hospital.
Hosp FE = hospital fixed effects; OR = odds ratio; SE = standard error.

© 2025 Hsuan C et al. *JAMA Network Open*.

eTable 6. Additional Analyses: Transfer if Initially Present at a Non–PCI-Capable Hospital

a. By Hour

	12-7 AM OR (SE)	8 AM-3 PM OR (SE)	4-11 PM OR (SE)
Transfer out, same/next day			
Hispanic	0.880	0.814*	0.705**
	(0.127)	(0.075)	(0.078)
Black non-Hispanic	0.720*	0.876	0.749*
	(0.113)	(0.093)	(0.087)
White non-Hispanic	[ref]	[ref]	[ref]
Other/Missing Race	0.644	0.615**	0.863
	(0.156)	(0.093)	(0.166)
self	0.731*	0.824*	0.756**
	(0.097)	(0.076)	(0.080)
Medicaid	0.803	1.120	1.173
	(0.138)	(0.136)	(0.162)
Medicare	1.772***	1.959***	1.790***
	(0.241)	(0.186)	(0.202)
Private	[ref]	[ref]	[ref]
Other/missing	1.001	0.965	0.944
	(0.272)	(0.169)	(0.211)
Observations	3,481	7,112	5,339

b. Weekend/Weekday

	Weekend OR (SE)	Weekday OR (SE)
Transfer out, same/next day		
Hispanic	0.815	0.773***
	(0.095)	(0.058)
Black non-Hispanic	0.823	0.789**
	(0.103)	(0.066)
White non-Hispanic	[ref]	[ref]
Other/Missing Race	0.676*	0.679**
	(0.130)	(0.088)
self	0.760*	0.782***
	(0.085)	(0.057)
Medicaid	1.146	1.018

© 2025 Hsuan C et al. *JAMA Network Open*.

	(0.170)	(0.097)
Medicare	2.037***	1.783***
	(0.242)	(0.136)
Private	[ref]	[ref]
Other/missing	0.799	1.053
	(0.189)	(0.151)
Observations	4,651	11,281

c. Excluding patients discharged against medical advice, alternative definition of transfers, controlling for number of STEMIs at initial hospital, controlling for number of hospitals in the hospital referral region, alternative definition of hospital

In the main analysis, we included patients discharged against medical advice because of concerns about bias. We include them in sensitivity analyses.

In the main analysis, transfers are defined as a same or next day visit. In the sensitivity analysis, we also required that the patient had a formal disposition code indicating transfer to another hospital or point of origin code at the next visit indicating transfer from another hospital.

We also controlled for the number of STEMIs at the initial hospital that year and for the number of hospitals within the hospital referral region (HRR) for that year. These two analyses might control for confounding if the initial (non-PCI-capable) hospital had more experience treating STEMI patients and had hospitals available to transfer patients to.

We also use an alternative definition of a PCI-capable hospital as one that was licensed by the state of Florida as an Adult Inpatient Diagnostic Cardiac Catheterization, Level I or Level II facility.

	Against Medical Advice	Disposition Code	# STEMIs	# hospitals in HRR	State licensed definition
	OR (SE)	OR (SE)	OR (SE)	OR (SE)	OR (SE)
Hispanic	0.778***	0.790***	0.788***	0.811**	0.778***
	(0.049)	(0.047)	(0.050)	(0.054)	(0.049)
Black non-Hispanic	0.785***	0.844**	0.794***	1.097	0.785***
	(0.055)	(0.056)	(0.055)	(0.081)	(0.055)
White non-Hispanic	[ref]	[ref]	[ref]	[ref]	[ref]
Other/Missing Race	0.658***	0.730**	0.675***	0.715**	0.658***
	(0.071)	(0.075)	(0.072)	(0.087)	(0.071)
self	0.798***	0.784***	0.774***	0.830**	0.798***
	(0.049)	(0.045)	(0.047)	(0.057)	(0.049)
Medicaid	1.065	0.958	1.047	1.044	1.065
	(0.086)	(0.071)	(0.084)	(0.089)	(0.086)
Medicare	1.900***	1.527***	1.833***	1.718***	1.900***

© 2025 Hsuan C et al. *JAMA Network Open*.

	(0.123)	(0.089)	(0.117)	(0.115)	(0.123)
Private	[ref]	[ref]	[ref]	[ref]	[ref]
Other/missing	0.994	1.025	0.966	0.973	0.994
	(0.124)	(0.119)	(0.118)	(0.119)	(0.124)
Observations	15,777	15,932	15,932	14,904	15,777

© 2025 Hsuan C et al. *JAMA Network Open*.

eTable 7. Additional Analyses: PCI Utilization at Receiving Hospital if Initially Present at a Non–PCI-Capable Hospital and Then Transferred

a. By Hour of ED Registration

We stratified analyses by the hour of ED registration (12-7 AM, 8 AM-3 PM, 4-11 PM)

	12-7 AM<br>OR (SE)	8 AM-3 PM<br>OR (SE)	4-11 PM<br>OR (SE)
Hispanic	0.750*	0.866	0.970
	(0.107)	(0.092)	(0.106)
Black non-Hispanic	0.549***	0.497***	0.570***
	(0.092)	(0.063)	(0.070)
White non-Hispanic	[ref]	[ref]	[ref]
Other/Missing Race	0.927	1.133	0.876
	(0.271)	(0.249)	(0.187)
self	1.056	0.854	0.733**
	(0.154)	(0.094)	(0.085)
Medicaid	0.633*	0.763	0.646**
	(0.117)	(0.109)	(0.092)
Medicare	0.682**	0.785*	0.542***
	(0.097)	(0.083)	(0.058)
Private	[ref]	[ref]	[ref]
Other/missing	0.814	0.844	0.737
	(0.215)	(0.180)	(0.157)
N	2,458	4,407	4,158

b. Weekend/Weekday

	Weekday-Overall<br>OR (SE)	Weekend-Overall<br>OR (SE)
Hispanic	0.868	0.936
	(0.068)	(0.118)
Black non-Hispanic	0.546***	0.522***
	(0.050)	(0.076)
White non-Hispanic	[ref]	[ref]
Other/Missing Race	1.020	0.881
	(0.162)	(0.212)
self	0.826*	0.920
	(0.067)	(0.126)
Medicaid	0.630***	0.818

© 2025 Hsuan C et al. *JAMA Network Open*.

	(0.065)	(0.135)
Medicare	0.647***	0.696**
	(0.051)	(0.089)
Private	[ref]	[ref]
Other/missing	0.789	0.784
	(0.118)	(0.206)
N	7,915	3,112

c. PCI Volume (# of PCIs conducted the previous year, by quartiles)

Since racial and ethnic minorities are more likely to go to hospitals that perform fewer PCIs, and hospitals that perform fewer PCIs might be less likely to perform PCIs, we stratified by PCI volume the prior year. Specifically, we calculated the number of PCIs each hospital performed the prior year and then calculated the quartile of PCIs conducted. Hospitals in quartile 1 performed the fewest PCIs the previous year; hospitals in quartile 4 performed the most PCIs the previous year.

We excluded quartile 1 from this analysis because of small sample size.

	Q1<br>OR (SE)	Q2<br>OR (SE)	Q3<br>OR (SE)	Q4<br>OR (SE)
Hispanic	0.903	0.908	0.844	0.905
	(0.141)	(0.118)	(0.132)	(0.106)
Black non-Hispanic	0.616*	0.553***	0.434***	0.536***
	(0.121)	(0.088)	(0.068)	(0.073)
White non-Hispanic	[ref]	[ref]	[ref]	[ref]
Other/Missing Race	1.269	0.806	1.075	1.094
	(0.428)	(0.209)	(0.286)	(0.283)
self	1.115	0.765	0.829	0.828
	(0.194)	(0.110)	(0.117)	(0.101)
Medicaid	0.682	0.632**	0.884	0.605**
	(0.141)	(0.110)	(0.170)	(0.093)
Medicare	0.515***	0.730*	0.717*	0.655***
	(0.087)	(0.100)	(0.099)	(0.075)
Private	[ref]	[ref]	[ref]	[ref]
Other/missing	0.716	1.107	0.763	0.638*
	(0.233)	(0.319)	(0.186)	(0.143)
N	1,749	2,663	2,742	3,817

d. Excluding 2020-2021 and using ICD9 and ICD10 years only

© 2025 Hsuan C et al. *JAMA Network Open*.

We excluded 2020-2021 from the analysis in case the COVID-19 pandemic, which disrupted ED use, changed PCI utilization (e.g. if Black non-Hispanic patients were more likely to delay going to the ED compared with White non-Hispanic patients). We also stratified analyses by whether the patient diagnoses were coded under ICD-9 or ICD-10 diagnosis codes.

	ICD9<br>OR (SE)	ICD10<br>OR (SE)	No COVID<br>OR (SE)
Hispanic	0.912	0.883	0.866*
	(0.085)	(0.084)	(0.061)
Black non-Hispanic	0.597***	0.480***	0.555***
	(0.062)	(0.056)	(0.045)
White non-Hispanic	[ref]	[ref]	[ref]
Other/Missing Race	1.071	0.890	1.006
	(0.198)	(0.171)	(0.142)
self	0.753**	0.968	0.840*
	(0.072)	(0.102)	(0.062)
Medicaid	0.653***	0.683**	0.680***
	(0.078)	(0.088)	(0.063)
Medicare	0.605***	0.733**	0.674***
	(0.056)	(0.071)	(0.048)
Private	[ref]	[ref]	[ref]
Other/missing	0.830	0.774	0.827
	(0.164)	(0.133)	(0.117)
N	5,897	5,128	9,766

e. **Including patients who died/transferred, excluding patients who left against medical advice, excluding patients who had diabetes, or including males only**

	Died/Transfer<br>OR (SE)	Against Medical Advice<br>OR (SE)	No Diabetes<br>OR (SE)	Men only<br>OR (SE)
Hispanic	0.914	0.892	0.888	0.851*
	(0.058)	(0.060)	(0.059)	(0.069)
Black non-Hispanic	0.556***	0.545***	0.541***	0.479***
	(0.041)	(0.043)	(0.042)	(0.046)
White non-Hispanic	[ref]	[ref]	[ref]	[ref]
Other/Missing Race	1.021	0.966	0.984	0.970
	(0.132)	(0.128)	(0.130)	(0.152)

© 2025 Hsuan C et al. *JAMA Network Open*.

self	0.825**	0.863*	0.845*	0.800**
	(0.056)	(0.061)	(0.059)	(0.065)
Medicaid	0.668***	0.703***	0.677***	0.687***
	(0.057)	(0.062)	(0.059)	(0.074)
Medicare	0.662***	0.669***	0.661***	0.660***
	(0.043)	(0.045)	(0.044)	(0.054)
Private	[ref]	[ref]	[ref]	[ref]
Other/missing	0.784	0.791	0.794	0.756*
	(0.098)	(0.104)	(0.103)	(0.107)
N	11,661	10,818	11,027	7,532

f. Urbanicity

	Large Central Metro OR (SE)	Large Fringe Metro OR (SE)	Medium Metro OR (SE)	Small metro, micropolitan & non-core OR (SE)
Hispanic	0.866	0.975	0.835	1.207
	(0.092)	(0.115)	(0.120)	(0.499)
Black non-Hispanic	0.582***	0.532***	0.468***	0.480
	(0.092)	(0.076)	(0.059)	(0.185)
White non-Hispanic	[ref]	[ref]	[ref]	[ref]
Other/Missing Race	0.838	1.079	1.082	0.873
	(0.190)	(0.268)	(0.271)	(0.510)
self	0.876	0.854	0.844	0.782
	(0.107)	(0.130)	(0.096)	(0.213)
Medicaid	0.691*	0.747	0.612***	0.716
	(0.105)	(0.144)	(0.088)	(0.232)
Medicare	0.738**	0.628**	0.622***	0.660
	(0.086)	(0.099)	(0.065)	(0.159)
Private	[ref]	[ref]	[ref]	[ref]
Other/missing	0.705	0.616	0.968	0.572
	(0.171)	(0.210)	(0.193)	(0.216)
N	3,581	2,258	4,186	995

g. Size of hospital

We stratified by the size of the receiving hospital. Only 757 patients were transferred to a hospital with fewer than 200 beds.

	200-500 beds	>500 beds

© 2025 Hsuan C et al. *JAMA Network Open*.

	OR (SE)	OR (SE)
Hispanic	0.971	0.803*
	(0.092)	(0.082)
Black non-Hispanic	0.500***	0.508***
	(0.056)	(0.061)
White non-Hispanic	[ref]	[ref]
Other/Missing Race	0.935	0.925
	(0.180)	(0.181)
self	0.768**	0.983
	(0.074)	(0.108)
Medicaid	0.738*	0.598***
	(0.088)	(0.085)
Medicare	0.605***	0.758**
	(0.055)	(0.081)
Private	[ref]	[ref]
Other/missing	0.638*	0.970
	(0.113)	(0.209)
N	5,928	4,287

h. Alternative definition of PCI-capable and hospital, and including all transfer patients who did not receive a PCI

In the main analysis, we used a threshold of 50 PCIs the prior year to define a hospital as PCI-capable. In sensitivity analyses, we used a threshold of 4 or more PCIs the prior year to define a hospital as PCI-capable. We also defined a PCI-capable hospital as one that was licensed by the state of Florida as an Adult Inpatient Diagnostic Cardiac Catheterization, Level I or Level II facility.

The main analysis defines hospital based on AHA hospital identifiers since we use AHA to define key variables including location. In the sensitivity analysis, we used the hospital identifier as defined by Florida.

In the main analysis, we defined the sample to be all transferred patients who initially presented to a non-PCI-capable hospital, and who did not receive a PCI. In the sensitivity analysis, we examined all patients who were alive and transferred, regardless of where they initially presented, who did not receive a PCI at the initial hospital.

	PCI4<br>OR (SE)	State-licensed<br>OR (SE)	Alt hosp def<br>OR (SE)	All transfer<br>OR (SE)
trt_pci				
Hispanic	0.890	1.024	0.906	0.970
	(0.061)	(0.080)	(0.059)	(0.058)
Black non-Hispanic	0.539***	0.499***	0.523***	0.544***

© 2025 Hsuan C et al. *JAMA Network Open*.

	(0.042)	(0.041)	(0.039)	(0.036)
White non-Hispanic	[ref]	[ref]	[ref]	[ref]
Other/Missing Race	0.964	0.951	1.032	0.906
	(0.131)	(0.141)	(0.135)	(0.100)
self	0.866*	0.837*	0.830**	0.872*
	(0.062)	(0.067)	(0.057)	(0.054)
Medicaid	0.687***	0.659***	0.685***	0.668***
	(0.061)	(0.064)	(0.059)	(0.052)
Medicare	0.690***	0.635***	0.651***	0.690***
	(0.047)	(0.048)	(0.043)	(0.040)
Private	[ref]	[ref]	[ref]	[ref]
Other/missing	0.801	0.780	0.801	0.905
	(0.107)	(0.107)	(0.102)	(0.100)
Observations	10,423	8,625	11,300	13,505

© 2025 Hsuan C et al. *JAMA Network Open*.

eTable 8. E Values

Outcome	Race/Ethnicity	E-value	E-value CI
Presentation to a PCI-capable hospital	Hispanic	2.787	1.975
	Black non-Hispanic	1.798	1.538
Receipt of PCI at PCI-capable hospital	Black non-Hispanic	1.913	1.838
Transfer if present to non-PCI-capable hospital	Hispanic	1.507	1.313
	Black non-Hispanic	1.491	1.272
Receipt of PCI at receiving hospital if initially present to non-PCI-capable hospital and transferred	Black non-Hispanic	2.056	1.832

© 2025 Hsuan C et al. *JAMA Network Open*.

References

1. Fl. Stat. Title 29 §395.1055
2. Lawton JS, Tamis-Holland JE, Bangalore S, Bates ER, Beckie TM, Bischoff JM, et al. 2021 ACC/AHA/SCAI Guideline for Coronary Artery Revascularization: Executive Summary: A Report of the American College of Cardiology/American Heart Association Joint Committee on Clinical Practice Guidelines. Circulation. 2022 Jan 18;145(3):e4-e17.

© 2025 Hsuan C et al. *JAMA Network Open*.
