# Supplementary material for: Disparities by Race and Ethnicity in Percutaneous Coronary Intervention
Source: JAMA Netw Open. 2025 Sep 18;8(9):e2532660. doi: 10.1001/jamanetworkopen.2025.32660 (PMC12447256; doi:10.1001/jamanetworkopen.2025.32660)
Supplement: Supplement 2. — Data Sharing Statement [file jamanetwopen-e2532660-s002.pdf]

Data Sharing Statement

Hsuan. Disparities by Race and Ethnicity in Percutaneous Coronary Intervention. *JAMA Netw Open*. Published September 18, 2025. doi:10.1001/jamanetworkopen.2025.32660

Data
Data available: No

Additional Information
Explanation for why data not available: The data are protected by a data use agreement that prohibits the authors from releasing it to others.
